# Gender-based vulnerability: combining Pareto ranking and spatial statistics to model gender-based vulnerability in Rohingya refugee settlements in Bangladesh

**DOI:** 10.1186/s12942-020-00215-3

**Published:** 2020-05-29

**Authors:** Erica L. Nelson, Daniela Reyes Saade, P. Gregg Greenough

**Affiliations:** 1 Harvard Humanitarian Initiative, Harvard University, Cambridge, MA USA; 2 Department of Emergency Medicine, Division of Global Emergency Care and Humanitarian Studies, Harvard Medical School, Brigham and Women’s Hospital, Boston, MA USA; 3 Graduate School of Geography, Clark University, Worcester, MA USA; 4 Harvard T.H. Chan School of Public Health, Boston, MA USA

**Keywords:** Rohingya, Refugees, Gender, Open-source data, Vulnerability index, Spatial analysis, GIS, Pareto ranking, Spatial autocorrelation

## Abstract

**Background:**

The Rohingya refugee crisis in Bangladesh continues to outstrip humanitarian resources and undermine the health and security of over 900,000 people. Spatial, sector-specific information is required to better understand the needs of vulnerable populations, such as women and girls, and to target interventions with improved efficiency and effectiveness. This study aimed to create a gender-based vulnerability index and explore the geospatial and thematic variations in gender-based vulnerability of Rohingya refugees residing in Bangladesh by utilizing pre-existing, open source data.

**Methods:**

Data sources included remotely-sensed REACH data on humanitarian infrastructure, United Nations Population Fund resource availability data, and the Needs and Population Monitoring Survey conducted by the International Organization for Migration in October 2017. Data gaps were addressed through probabilistic interpolation. A vulnerability index was designed through a process of literature review, variable selection and thematic grouping, normalization, and scorecard creation, and Pareto ranking was employed to rank sites based on vulnerability scoring. Spatial autocorrelation of vulnerability was analyzed with the Global and Anselin Local Moran’s I applied to both combined vulnerability index rank and disaggregated thematic ranking.

**Results:**

Of the settlements, 24.1% were ranked as ‘most vulnerable,’ with 30 highly vulnerable clusters identified predominantly in the northwest region of metropolitan Cox’s Bazar. Five settlements in Dhokkin, Somitapara, and Pahartoli were categorized as less vulnerable outliers amongst highly vulnerable neighboring sites. Security- and health-related variables appear to be the most significant drivers of gender-specific vulnerability in Cox’s Bazar. Clusters of low security and education vulnerability measures are shown near Kutupalong.

**Conclusion:**

The humanitarian sector produces tremendous amounts of data that can be analyzed with spatial statistics to improve research targeting and programmatic intervention. The critical utilization of these data and the validation of vulnerability indexes are required to improve the international response to the global refugee crisis. This study presents a novel methodology that can be utilized to not only spatially characterize gender-based vulnerability in refugee populations, but can also be calibrated to identify and serve other vulnerable populations during crises.

## Background

The Rohingya refugee crisis is a result of decades of systematic discrimination, statelessness, and violence exacerbated by the denaturalization of the Rohingya people by the then-Burmese state in 1977 [1]. Since that time, waves of Rohingya, now considered ‘illegal’ in their birth country, have fled into Bangladesh in a complex cycle of forced displacement and repatriation efforts [2]. In August of 2017, violent attacks perpetrated against the Rohingya triggered the largest and fastest mass displacement of refugees from Myanmar’s Rakhine State to Bangladesh. As of March of 2019, there were over 909,000 Rohingya refugees in Ukhiya and Teknaf upazilas, representing an influx of over 745,000 people in less than 2 years [3].

Women and girls comprise over 52% of the population in Rohingya settlements in Bangladesh, with approximately one-sixth of families headed by a single mother [4]. Almost every woman and girl in the Rohingya refugee community in Cox’s Bazar has either experienced or witnessed incidences of gender-based violence [5], and the crisis disproportionately affects women, girls, and other marginalized populations due to the perpetuation of pre-existing inequalities, violence, and discrimination. As of February 2018, the Inter-Agency Working Group on Reproductive Health in Crisis [6] identified women and girls as ‘critically underserved’ in the Rohingya Humanitarian Response, emphasizing a lack of access to sexual and reproductive services and gender-based violence care. According to the World Food Program, Rohingya refugee households in Bangladesh headed by women are disparately more vulnerable to food insecurity, with 45% defined as vulnerable or highly vulnerable [7]. Over one-third of Rohingya women report insecurity when collecting water or toileting [8], and over half of the female population lack appropriate menstrual supplies. Many women remain within their shelters due to a lack of clothing, insecurity, and concerns regarding cultural norms and dignity. Despite the humanitarian community’s commitment to gender mainstreaming and women-targeted strategies [9] in response efforts, qualitative data from a 2018 Oxfam report demonstrated that the humanitarian response has yet to adequately meet the needs of women and girls in this community, failing to provide them with access to services and/or addressing gender-specific issues critical to preventing further harm [4].

As is the case in many humanitarian and disaster response environments, the sheer magnitude of the Rohingya crisis juxtaposed with nearly 272 million United States Dollars (USD) in unmet funding requirements [10] compels donors and humanitarian actors to create mechanisms to more precisely characterize need and design targeted, cost-effective services. One such method of need assessment is through the lens of ‘vulnerability’ analysis, which articulates the reality that hazards and subsequent assistance impact various population groups in grossly heterogeneous ways [11]. Numerous vulnerability indices have emerged in fields including infectious diseases [12, 13], environmental health [14, 15], disaster preparedness [16–18], refugee services [7, 19], and climate change [20–22] which incorporate diverse, cross-sectoral indicators such as socio-economic status, education, information access, mobility, health and morbidity, security, and geographic location.

Such vulnerability indices require significant data, which is often incomplete, untimely, and ambiguous in the humanitarian context. Designing and implementing primary data collection studies in humanitarian settings requires substantial resources that can be otherwise utilized for response activities and is further complicated by insecure or inaccessible environments. However, there exist tremendous data being produced by agencies already operating in these contexts, and a recent impetus to share these invaluable data has led to open source dissemination platforms. The Humanitarian Data Exchange (HDX) [23], managed by United Nations Office for the Coordination of Humanitarian Affairs’ (OCHA) Center for Humanitarian Data, was launched just over 5 years ago and is an online, open source data platform aimed at making humanitarian data available for analysis and use by non-governmental organizations, governments, and United Nation agencies. As of April 2018, it houses over 6000 data sets from nearly 1000 sources in 245 locations [24].

The aim of this study was to harness the potential of pre-existing, open source data provided by the Humanitarian Data Exchange to create a gender-based vulnerability index and explore the geospatial and thematic variations in the gender-based vulnerability of Rohingya refugees residing in Bangladesh.

## Methods

This study designed a gender-based vulnerability analysis grounded in constructs derived from a literature review and then modified due to the constraints of data availability, executed a Pareto ranking as a method to avoid artificial weighting in aggregated index scores, and subsequently employed methods of spatial autocorrelation and cluster/outlier analysis. This methodological framework was applied to open-source humanitarian data collected at Rohingya refugee settlements in Bangladesh directly after the 2017 refugee influx. Briefly, this methodology intends to capitalize on pre-existing spatial humanitarian data to identify those refugee settlements wherein women and girls are the most vulnerable, and create a statistically-grounded, spatial understanding of gender-based vulnerability that can lead to more relevant and better targeted programmatic design and resource allocation.

### Data collection

Three data streams were utilized for this study, including the International Organization for Migration (IOM) Needs and Population Monitoring Data from October 2017, in Bangladesh; REACH geocoded infrastructure location data, and United Nations geocoded resource availability data. These data were obtained from the open source, online Humanitarian Data Exchange between October 2017 and January 2018. A description of these data and data collection methodologies follow.

The International Organization for Migration conducted a Needs and Population Monitoring site assessment of Rohingya refugee communities at 28 collective sites and 99 locations within dispersed settings in host communities in Cox’s Bazar, Bangladesh between the 30th of September and 9 October 2017 [25] with a final sample size of 157 sub-site assessments. The site assessment covers many locations where Rohingya refugees have been identified, notwithstanding the type of location or proximity to Bangladeshi host communities, but is not a comprehensive survey of all refugee sites. Information regarding the demographics, distribution, and needs of the Rohingya refugee population spanned 12 areas of inquiry and 184 unique questions that were both quantitative and qualitative in nature. Information was collected by locally-recruited enumerators through key informant interviews using closed-ended questionnaires regarding indicators such as demographics, access to information, services and resources, infrastructure, security and protection parameters, and disease outbreak. Findings were triangulated at the field level through direct observation and spontaneous group discussions. Indicators utilized in this study were derived directly from this survey and its respondents’ interpretation of the survey questions (e.g. respondents were not given specific definitions of ‘barriers of access to education’ or ‘enough water for household needs’).

This study utilized both REACH and United Nations Population Fund (UNFPA) data streams that provided seven datasets regarding locations of resources [26–32]. These datasets included geo-coded surveys that identified health care facilities, child- and women-friendly spaces, and distribution centers that were conducted between November 2017 and February 2018. These data, comprised of either geodatabases containing point data, shapefiles, and/or spreadsheets containing each facility’s spatial coordinates, were incorporated into a larger geodatabase, joined spatially, and cleaned for later analysis. Distance to distribution centers, health care, nutritional facilities, and child- and women-friendly spaces were determined by identifying resource locations from previously described datasets and measuring the distance to the centroid of the settlement utilizing Euclidean distances.

### Gender-based vulnerability index: conceptual framework and indicators

This study followed a multi-step process for developing a gender-based vulnerability score, founded on principles articulated in the Handbook on Constructing Composite Indicators [33]. After reviewing existing literature, a theoretical framework was developed to serve as a basis for selecting indicators. By reviewing pre-existing data available through the HDX database, component themes and indicators were identified to include in the gender-based vulnerability index (Table 1).

**Table 1:** Component themes and indicators selected to contribute to the gender-based vulnerability index

Component theme	Indicators	Source
	Indicators derived from the IOM survey include a description of the question asked, codification of responses, and determination of relative vulnerability in this study Indicators derived from alternate sources include a description of how that indicator was measured and the determination of relative vulnerability in this study	
Demographics	Percentage of pregnant women. Increasing percentages were considered ‘more vulnerable’. Numerical score reported as a decimal (0–1)	IOM Survey
	Percentage of women lactating. Increasing percentages were considered ‘more vulnerable’. Numerical score reported as a decimal (0–1)	IOM Survey
Education	Presence of barriers to education for adolescent girls: ‘Are there any barriers to accessing education for adolescent girls?’ Responses were codified as ‘yes’, ‘no’, and ‘don’t know’ Presence of barriers to education was defined as ‘more vulnerable’. Numerical score = 1 if barriers were present	IOM Survey
Health	Access to antenatal care: ‘Do people face problems accessing antenatal care?’ Responses were codified as ‘yes’, ‘no’, ‘no access’, and ‘don’t know’ In this study, problems accessing antenatal care and ‘no access’ were considered equally ‘more vulnerable’. Numerical score = 1 if ‘no access’ or ‘problems accessing’	IOM Survey
	Access psychosocial support services: ‘Do people face problems accessing psychosocial support?’ Responses were codified as ‘yes’, ‘no’, ‘no access’, and ‘don’t know’ In this study, problems accessing psychiatric support services and ‘no access’ were considered equally ‘more vulnerable’. Numerical score = 1 if ‘no access’ or ‘problems accessing’	IOM Survey
	Access to vaccinations: ‘Do people face problems accessing vaccination services?’. Responses were codified as ‘yes’, ‘no’, ‘no access’, and ‘don’t know’ In this study, problems accessing vaccinations and ‘no access’ were considered equally ‘more vulnerable’. Numerical score = 1 if ‘no access’ or ‘problems accessing’	IOM Survey
	Distance to the nearest healthcare facility: Healthcare facilities’ locations were identified, and Euclidean distance between the nearest facility and the settlement site were determined in ArcGIS ‘Vulnerability’ was considered to increase with distance to facility. Score = distance/max distance as described in the narrative	REACH and UNFPA
Water, Sanitation, and Hygiene (WASH)	Perception of ‘enough water for household needs’: ‘Is there enough water to meet household needs in this settlement/camp?’ Responses were codified as ‘yes’, ‘no’, and ‘don’t know’ Those camps/settlements without water to meet household needs were classified as ‘more vulnerable’. Numerical score = 1 if ‘without water to meet needs’	IOM Survey
	Recent outbreaks of diarrhea: ‘Have there been outbreaks of diarrhea, recently?’ Responses were codified as ‘yes’, ‘no’, and ‘don’t know’ Those camps/settlements with recent outbreaks of diarrhea were classified as ‘more vulnerable’. Numerical score = 1 if recent outbreak	IOM Survey
	Where women defecate at night: Responses codified as ‘private facilities’, ‘communal’, and’ open defecation’ In this study, ‘open defecation’ was defined as the most ‘vulnerable’ (score = 2), with ‘communal’ defined as ‘less vulnerable’ (score = 1), and ‘private facilities’ as ‘least vulnerable (score = 0)	IOM Survey
Resource Availability	Access to food supplementation for pregnant and lactating women: ‘Does your community face problems/challenges accessing nutritional supplements for pregnant and lactating women?’ Responses were codified as ‘supplement service available, but there are problems accessing it’, ‘no problem accessing supplements’, ‘no supplement service available’, ‘don’t know’ Communities with ‘problems accessing’ supplementation and ‘no supplement service available’ were defined by this study as equally ‘more vulnerable’. Numerical score = 1 if ‘no services available’ or ‘problems accessing’.	IOM Survey
	Distance to the nearest distribution center: Distribution centers’ locations were identified and Euclidean distance between the nearest facility and the settlement site were determined in ArcGIS ‘Vulnerability’ was considered to increase with distance to facility. Score = distance/max distance	REACH and UNFPA
Security	Access to gender-based violence services: ‘Is there access to gender-based violence services?’ Responses were ‘yes’, ‘no’, and ‘don’t know’ Communities with no access to gender-based violence services were considered ‘more vulnerable’. Numerical score = 1 if ‘no access’	IOM Survey
	Access to incident reporting mechanism: ‘Is there an incident reporting mechanism?’ Responses were ‘yes’, ‘no’, and ‘don’t know’ Communities with no incident reporting mechanism were considered ‘more vulnerable’. Numerical score = 1 if ‘no mechanism’	IOM Survey
	Access to police and courts: ‘Do people in your community have access to police and courts?’Responses were ‘yes’, ‘no’, and ‘don’t know’ No access to police and courts was considered ‘more vulnerable’. Numerical score = 1 if ‘no access’	IOM Survey
	Distance to the nearest ‘women-friendly space’, which is defined as a space in which women and girls feel physically and emotionally safe [34]. ‘Women-friendly space’ locations were identified from the data source, and Euclidean distance between the nearest facility and the settlement site were determined in ArcGIS ‘Vulnerability’ was considered to increase with distance to facility. Score = distance/max distance	REACH
	Distance to the nearest ‘child-friendly space’, which is defined as a place to support and protect children with the objective to restore a sense of normalcy [35]. ‘Child-friendly space’ locations were identified from the data source, and Euclidean distance between the nearest facility and the settlement site were determined in ArcGIS ‘Vulnerability’ was considered to increase with distance to facility. Score = distance/max distance	REACH

All distances are in kilometers

Vulnerability, commonly defined in the humanitarian community as the diminished capacity of an individual or group to anticipate, cope with, and resist and recover from the impact of a hazard [11], is a relative and dynamic concept. It is multi-dimensional and differential, scale-dependent, and spatially heterogeneous. The definition of vulnerability suggests that it is referential to specific hazards. In the setting of Rohingya refugees, hazards are abundant, including vulnerability to food insecurity, interpersonal violence, diseases, and natural hazards such as cyclones, landslides, and flooding. In light of such reality, this study defines vulnerability in alignment with the Vulnerability Assessment Framework established by the United Nations High Commissioner for Refugees as the ‘risk of exposure of … refugee households to harm, primarily in relation to protection threats, inability to meet basic needs, limited access to basic services, and food insecurity, and the ability of the population to cope with the consequences of this harm” [19].

Vulnerability is frequently differentiated by gender. Variables, such as lack of access to and/or control over essential resources and lack of entitlements, compound women’s vulnerability and undermine agency and adaptive and recovery capacities from hazards [36]. Many gender indices exist to examine the role of gender discrimination as it influences human development and wellbeing at the national level. These indices, including the Social Institutions Gender Index [37], the Gender Inequality Index [38], the Global Gender Gap Index [39], identify several dimensions that influence gender inequality. These range from health and protection parameters such as access to prenatal care and laws regarding gender-based violence, attainment of education, economic indicators, and freedom of movement, to discriminatory family codes, restricted civil liberties, representation in government, and empowerment within socio-economic realms. Although more specifically targeting a development context, these gender indices, along with the Plan India gender vulnerability index [40], provided conceptual, validated guidance on which indicators reported in the available, open-source datasets are most accurate in predicting gender-based vulnerability.

### Addressing incomplete data with probabilistic interpolation

Within the IOM’s Needs and Population Monitoring dataset, there were assessment points that lacked data regarding specific indicators. For instance, gaps in gender-specific food insecurity coping strategies such as preferentially feeding boys over girls were not often included. Rather than include these incomplete data, a more spatially-relevant indicator of access to food at distribution points was utilized given these were more complete in the dataset. Indicators marked as ‘I don’t know’ by the respondent were considered ‘missing data’. All of the indicators used in the index were complete for the settlements included in our analysis with the exception of access to police and courts (three missing values) and recent diarrhea outbreaks (two missing values). For these two binary indicators, we assumed spatial randomness within the geographic bounds of the data and imputed values by assigning likelihoods of “0” or “1” or using a probabilistic interpolation method of indicator formalism [41]. This non-parametric method required transforming the missing binary value to a probability for that value taking into account the indicator’s value at other locations. The probability of a binary value for a given indicator, *I*, at a location *u,* for a binary or categorical value *z*_*k*_ is the transformation:

$$I\left( {u:z_{k} } \right) = \left\{ {1, \, if\;Z\left( u \right) = z_{k} } \right\} \,or \,\left\{ {0, \, if\;otherwise} \right\}$$

Several sites possessed so little data they could not be included in the analysis, making the final number of settlements evaluated 145.

### Quantifying and normalization

#### Quantification

Each variable was enumerated for incorporation into the vulnerability scoring, with higher scores indicating a higher contribution to vulnerability. Non-enumerated variables, such as binary variables that defined the presence or absence of service, were quantified as vulnerable = 1 and not vulnerable = 0. For example, if prenatal care was available, the site would be scored a zero, and if prenatal care was not available, the site was scored a one, given the assumption that a failure to provide prenatal care would make women and girls more vulnerable. Similarly, the places in which women defecate at night were reported as a generalized response of either (1) in private, (2) in communal latrines, or (3) open defecation. These were scored as 0–2, with open defecation defined as the most vulnerable. Further clarification regarding those indicators that were defined as ‘more vulnerable’ verses ‘less vulnerable’ and the subsequent numerical scores are provided in Table 1.

Distance to facilities or resources were measured in kilometers (Km) utilizing Euclidean distance and quantified within the scorecard with the equation:

$$Score = Distance\, \left( {\text{km}} \right)\;to\;facility/Max\;distance\, \left( {\text{km}} \right)$$

thereby creating a vulnerability score that is relative to other refugee sites. Variables that were reported as percentiles (e.g., percent lactating women) were incorporated as decimals, normalizing the variable between zero and one.

Within each thematic component of the vulnerability index (e.g. health, sanitation, security, etc.), the variables within that theme were summed to create an aggregate thematic vulnerability score and were normalized utilizing the methodology, below.

#### Normalization

Each thematic score was normalized to prevent arbitrary weighting of individual thematic components utilizing the equation below, which has been employed by many studies and is recommended by the Humanitarian Development Index as a standardized method [42].

$$Normalized\;score = \frac{{\left( {x - x_{min} } \right)}}{{\left( {x_{max} - x_{min} } \right)}}$$

### Aggregating thematic component scores and the role of Pareto ranking

Composite vulnerability indices have long been criticized for employing arbitrary calculations to aggregate indicator scores into a single metric [43]. Thematic component scores (e.g., health or security) may be simply averaged or a weights-matrix may be applied, but both of these mathematical decisions are problematic and more likely represent the researcher’s biases than the realities of vulnerability. Simple averaging obscures extreme values when both high and low scores exist in different thematic components. Weighting schema are equally problematic, requiring researchers to judge and quantify the relative importance of different components either through Delphi procedures or qualitative or quantitative methods. Even qualitatively derived weights matrices will likely fail the test of external validation, as the importance of indicators is spatio-temporally heterogeneous.

Thus, this study utilized Pareto ranking, as implemented by Rygel et al. [44], to avoid the aforementioned challenge of aggregation and the subjective weighting of thematic components, and yet still create a composite vulnerability hierarchy that takes into account multiple variables. While this produces only a relative understanding of vulnerability within a study area, it was deemed appropriate given that the aims of this study were to identify the ‘most vulnerable’ communities for resource allocation.

**Fig. 1:**
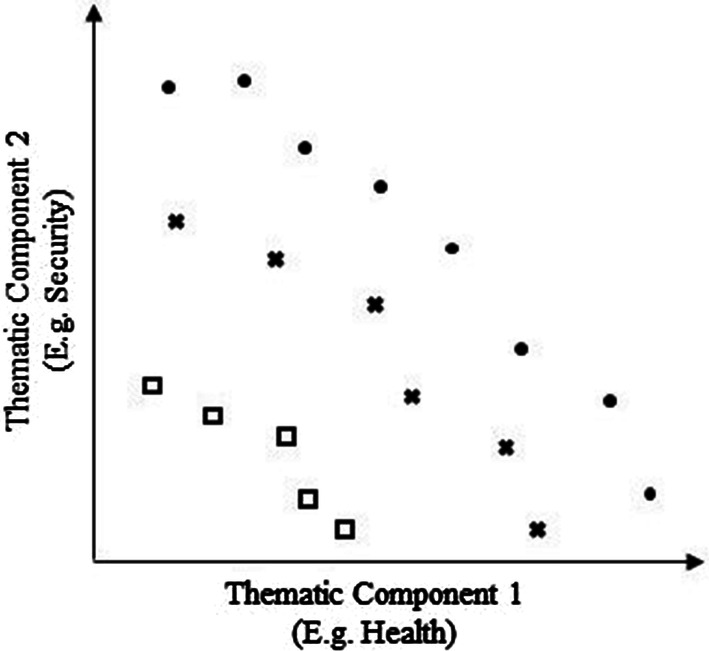
A two-dimensional illustration of Pareto ranking in which each data point (e.g. settlement site) has two thematic component scores (represented on X and Y axes). Those data points represented as circles are considered the most vulnerable rank, in that each site is non-dominated. Those represented as ‘X’s are second-most vulnerable, and those represented by squares are least vulnerable

Pareto ranking is a method by which non-domination is applied to ascribe vulnerability ranks to each refugee site. Non-domination is achieved by a site when no other sites in the dataset are more vulnerable, i.e., if in each thematic component, that site scores at least as high or higher than all other refugee sites. The non-dominated set of sites includes all sites that are non-dominated in the dataset and represent the most vulnerable. These sites are then removed from the dataset, and the algorithm iterated to identify the next most vulnerable set of refugee sites. The process is repeated until all sites are ranked, thereby creating a hierarchical ranking of gender-based vulnerability amongst these sites. An illustration of this process is provided in Fig. 1. Note that this illustration demonstrates each point as having only two thematic components that factor into its vulnerability ranking. In this study, Pareto ranking utilized the six thematic components identified above with identical logic and procedures applied to the higher-dimensional data.

The Pareto ranking algorithm was executed in Python with researcher-created code.

### Geospatial analysis

Spatial analysis has been extensively adopted for exploring spatial heterogeneity and cluster detection in public health research and environmental epidemiology [45–47] Ranging greatly in application and methodology, spatial autocorrelation and local indicators of spatial association (LISA) have been utilized to appreciate clusters of disease, morbidity, and mortality, and thereby contribute to a knowledge base surrounding exposure pathways, social determinants of health, and the intersection between socio-economic, environmental, and human wellbeing. Practically, cluster detection methods have provided a means by which health policy officials and public health personnel can better target interventions and further research. Through this lens of spatial epidemiology, this study undertook a geospatial analysis of gender-based vulnerability ranking to characterize the spatial heterogeneity that would lead to a better spatial understanding of gender-based vulnerability in this community and could potentiate better targeting of programs for women and girls within the Rohingya refugee settlements. Spatial autocorrelation analysis and LISA, utilizing the Global Moran’s and Anselin Local Moran’s I, were employed to identify large-scale trends of autocorrelation and clusters and outliers of refugee settlement sites that possessed high and low gender-based vulnerability scores.

Global Moran’s I is an inferential, spatial statistical method that determines spatial autocorrelation based on feature location and attributes value, e.g., vulnerability score utilizing the equation:

$$I = \frac{N}{W}\frac{{\varSigma i \varSigma j w_{ij } \left( {x_{i} - \bar{x}} \right) \left( {x_{j } - \bar{x}} \right)}}{{\varSigma_{i } \left( {x_{i } - \bar{x}} \right) ^{2} }}$$

Wherein *N* is the number of spatial units indexed by *i* and *j*, *x* and *xbar* are the variables of interest and its mean, *w*_*ij*_ is a matrix of spatial weights, and *W* is the sum of all *w*_*ij*_. With the null hypothesis being no spatial autocorrelation, the expected value of Moran’s I is

$$E\left( I \right) = \frac{ - 1}{N - 1}$$

Wherein, as *N* approaches infinity, the expected value approaches zero [48]. Application of the Global Moran’s I statistic allows for the characterization of the pattern of features and associated attributes to be identified as clustered, dispersed, or random. Positive values of Moran’s I with significant p-values suggest clustering of features more than expected given complete spatial randomness.

Anselin’s Local Moran’s I was then executed to characterize clusters of settlements that possessed high and low gender-based vulnerability scores and to identify those communities that had scores statistically higher or lower than neighboring settlements, i.e., outliers. The calculations below were applied to both the Pareto ranks as an aggregate gender-based vulnerability score and subsequently to disaggregated thematic scores to lend insight into which thematic variables, such as health or security, are potentiating high/low levels of vulnerability in those communities.

Local Moran’s I statistic [49] of spatial association is given as:

$$I_{i } = \frac{{x_{i} - \bar{X}}}{{S_{i}^{2} }}\mathop \sum \limits_{j = 1, j \ne i}^{n} w_{i,j} \left( {x_{j } - \bar{X}} \right)$$

Where *X*_*i*_ is an attribute for feature *i*, *Xbar* is the mean of the corresponding attribute, *w*_*i,j*_ is the spatial weight between feature *i* and *j*, and

$$S_{i}^{2} = \frac{{\mathop \sum \nolimits_{j = 1, j \ne i}^{n} \left( {x_{j} - \bar{X}} \right) ^{2} }}{n - 1}$$

with *n* equal to the total number of features.

In this study, parameterization of the model included the adoption of inverse distance weighting as the spatial conceptual model, Euclidean distance as the measurement construct, and row standardization to address any sampling bias present in the IOM settlement assessments. Inverse distance weighting was the spatial relationship chosen given the spatial heterogeneity of settlements and to avoid the arbitrary nature of choosing the number of nearest neighbors or distance thresholds in alternate weighting matrix parameters. These parameters were applied throughout both Global Moran’s I and Anselin’s Local Moran’s I analyses.

All mapping and spatial analyses were done within ESRI ArcMap 10.6 software. The study was reviewed by the Harvard T.H. Chan School of Public Health Institutional Review Board and determined to be exempt from full review given that it involves open-source data and is not explicitly human subjects research.

## Results

### Pareto ranking of gender-based vulnerability

Only 145 of the 174 settlements sampled by the IOM’s Needs and Population Monitoring Survey possessed enough data to be included in the vulnerability analysis. The Pareto rankings determined by this methodology ranged from 1 to 6, with one being the least vulnerable and six being the most vulnerable (Table 2). Approximately 24%, or 35 settlements, were deemed ‘most vulnerable’ based upon Pareto rankings of the thematic elements. Thirty-nine settlements were characterized as the second most vulnerable, comprising 26.9% of the refugee communities. Only one settlement was considered ‘least vulnerable,’ and 15 settlements were considered second to least vulnerable, comprising 10.3% of the analyzed encampments. The remaining 55 settlements have intermediate scores of relative gender-based vulnerability.

**Table 2:** Outcome of Pareto ranking of gender-based vulnerability analysis, including the absolute number of settlements and percent of all sites evaluated

	Least vulnerable					Most vulnerable
	1	2	3	4	5	6
Number of settlements	1	15	29	26	39	35
Percentage (%)	< 1	10.3	20	17.9	26.9	24.1

**Fig. 2:**
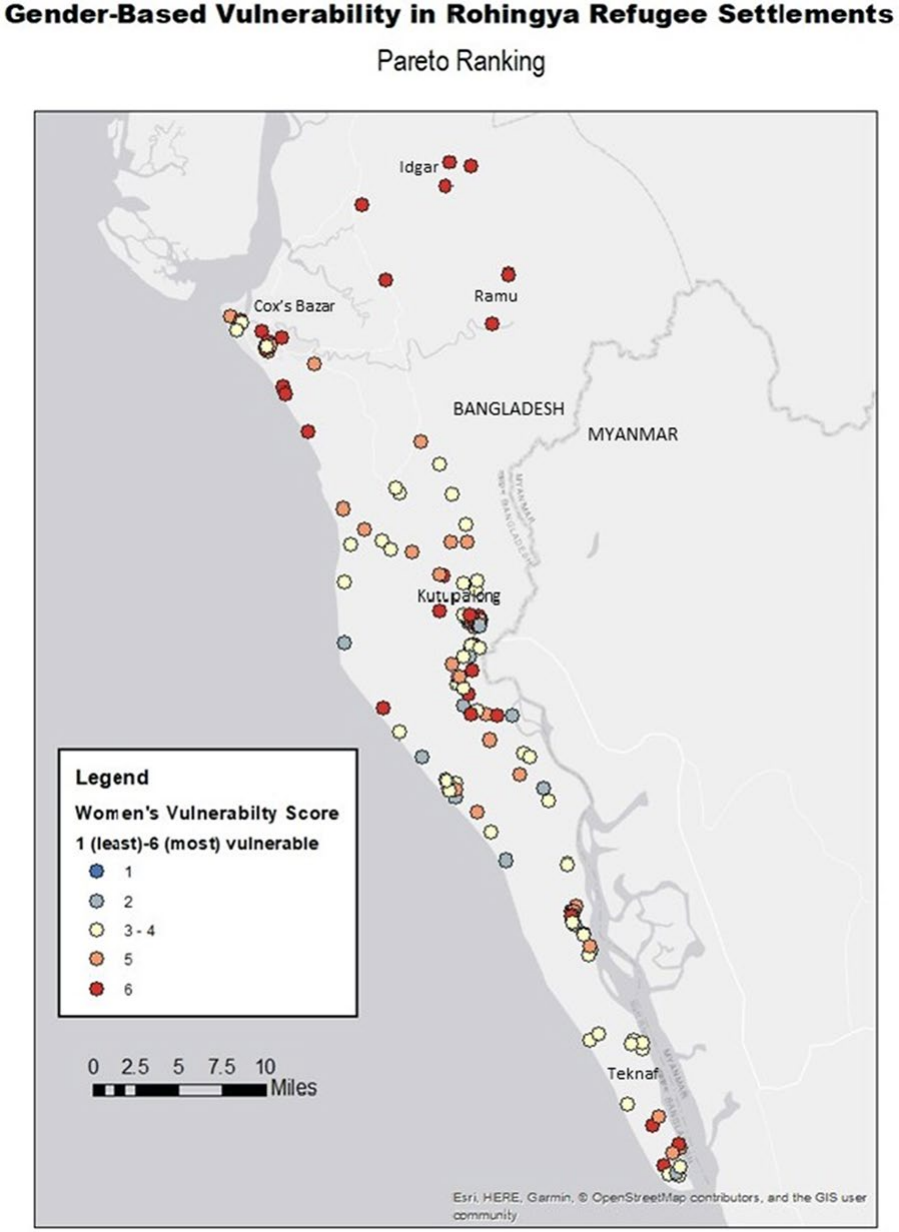
Total gender-based vulnerability index rankings of Rohingya refugee settlements in Bangladesh wherein 1 = least vulnerable and 6 = most vulnerable

The spatial distribution of the relative gender-based vulnerability scores can be seen in Fig. 2. Grossly, a large geographic spread of gender-based vulnerability is evidenced throughout the study area. Visuo-spatial patterns identify higher levels of vulnerability around Cox’s Bazar and the north of the region, along the Myanmar/Bangladesh border near Gundam, and in the south-east near Teknaf. Those settlements with the lowest gender-based vulnerability appear to be dispersed, but predominantly located in the south-east of the region, with intermediate scores predominating much of Ramu upazila.

This visuo-spatial intuition of the clustering of higher levels of gender-based vulnerability is ratified by the Global Moran’s I spatial autocorrelation analysis in which spatial clustering is significantly noted. With a Z-score of 4.5 and a P-value less than 0.001, the spatial distribution of high and low vulnerability scores demonstrate a spatial clustering more than would be expected given complete spatial randomness.

**Fig. 3:**
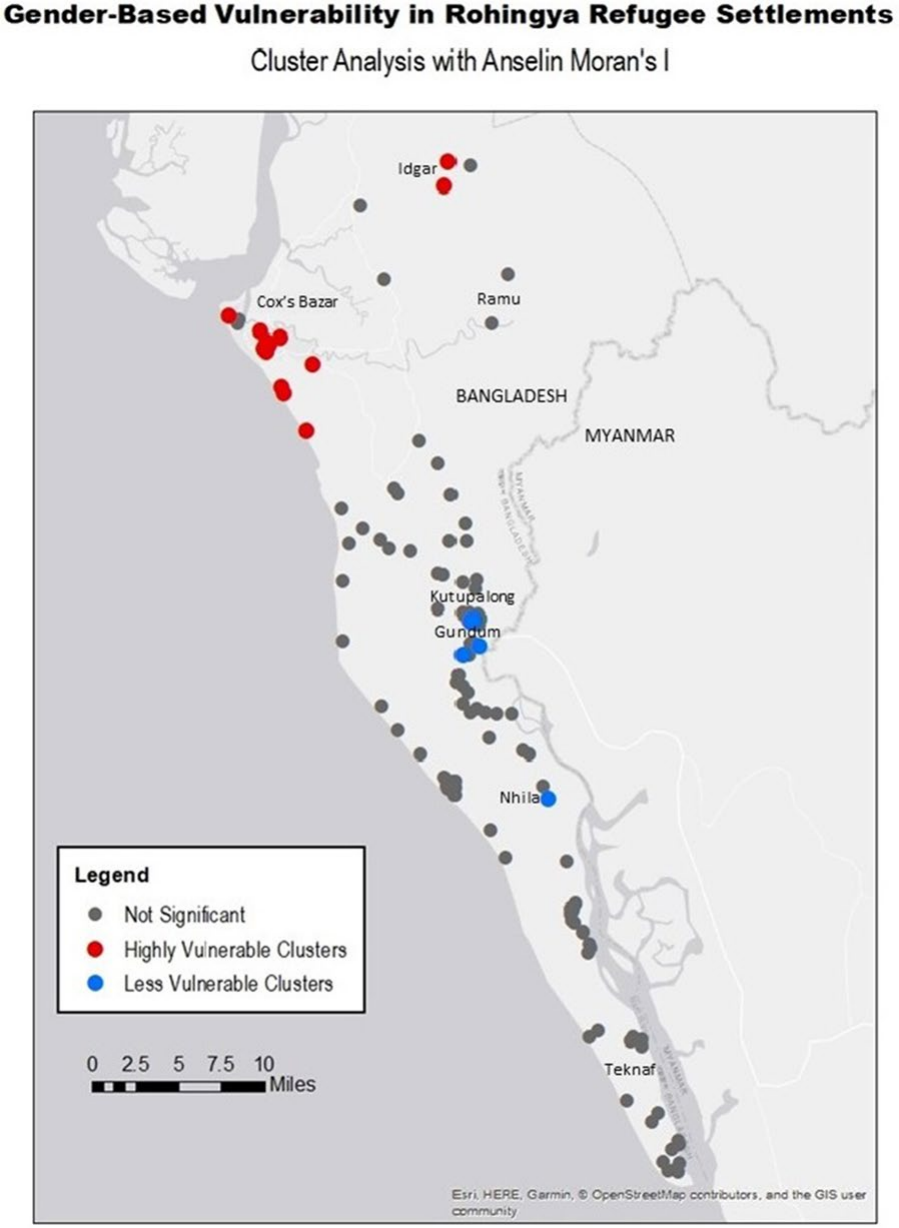
Cluster analysis with Anselin Local Moran’s I of gender-based vulnerability in Rohingya Refugee settlements in Bangladesh

The Anselin Local Moran’s I analysis demonstrated thirty settlements that occupy ‘highly vulnerable’ clusters, with only five settlements comprising ‘less vulnerable’ clusters (Table 3). Several communities in the northwest of the study area (Fig. 3) around Cox’s Bazar, delineated as statistically-significant, ‘highly vulnerable’ clusters are, strictly speaking, the cores of each ‘highly vulnerable’ cluster. Two settlements in Idgar, along the northernmost part of the study region, are spatially disparate cores of two high-vulnerability clusters. Less vulnerable clusters are demonstrated in the south-east region near Kutupalong, its expansion and surrounding settlements, and near Dakshin Nhila.

**Table 3:** Outcome of Anselin’s Local Moran’s I analysis of gender-based vulnerability ranking characterizing both clusters and outliers

Analysis outcome	Number of sites
Highly vulnerable clusters	30
Highly vulnerable outliers	8
Less vulnerable outliers	3
Less vulnerable clusters	5
Not significant	115

**Fig. 4:**
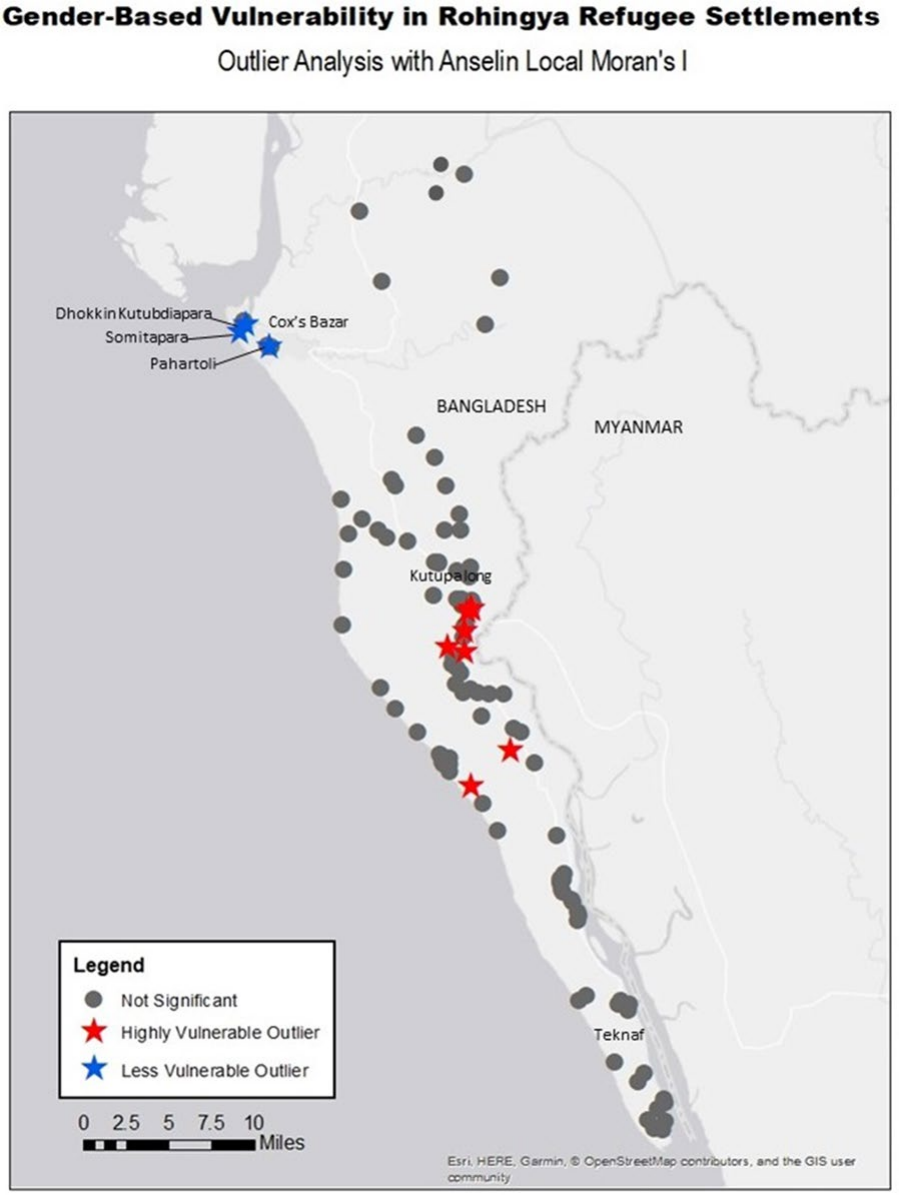
Outlier analysis with Anselin Local Moran’s I of gender-based vulnerability in Rohingya Refugee settlements in Bangladesh

Outlier communities that display statistically significant higher or lower vulnerability scores than their neighbors are demonstrated in Fig. 4. Given the evidenced generalization that gender-based vulnerability is higher in the Cox’s Bazar metropolitan region, the outcomes of the outlier analysis are justified, with all low-level vulnerability outliers, the settlements of Dhokkin Kutubdiapara, Somitapara, and Pahartoli, existing in that vicinity. Conversely, those settlements that are statistically more vulnerable than surrounding neighbors reside within the relatively less vulnerable regions along the Myanmar/Bangladesh border near Kutupalong.

**Fig. 5:**
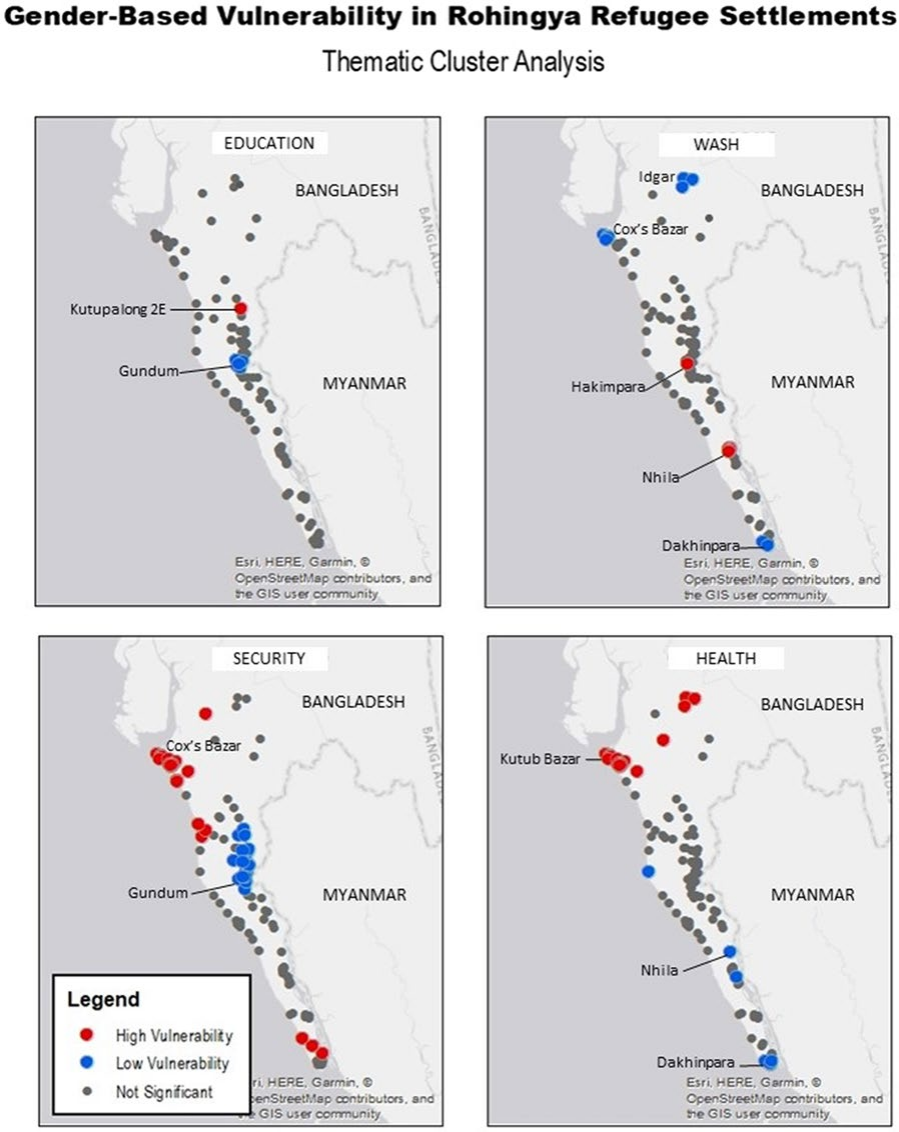
Cluster analysis of gender-based vulnerability in Rohingya refugee settlements as disaggregated by thematic components, including education, WASH, security, and health

When disaggregated into thematic components of education, WASH, security, and health, the spatial heterogeneity of hot- and cold-spots of gender-based vulnerability remains (Fig. 5). Gender-based health and security variables display significant spatial dependence. Those previously classified, composite highly-vulnerable clusters in the northwestern regions near the metropolitan area of Cox’s Bazar are now defined as the most-vulnerable settlements when considering health and security indicators. Conversely, security-dependent, gender-based vulnerability is lowest in refugee settlements along the border with Myanmar. And refugee settlements identified as the centroids of low levels of health-related vulnerability clusters are in the south-east, and specifically in Nhila and Dakshinpara along the Naf River. Education variables are demonstrated to have very few statistically significant clusters, yet educational access appears to be best along ingress points near Gundam and problematic at the Kutupalong 2E refugee site. Water, sanitation, and hygiene clusters are similarly sparse, but cold-spots are identified in the most northern and southern refugee settlements, and in the Kutub Bazar community near Cox’s Bazar, where composite least vulnerable outliers were demonstrated previously.

## Discussion

Through the application of a vulnerability index and subsequent spatial analytics to open source humanitarian data, this study demonstrated significant spatial heterogeneity of gender-based vulnerability in Rohingya refugee settlements in the southwest of Bangladesh. Clusters of high levels of gender-based vulnerability were identified in or around the town of Cox’s Bazar, with clusters of low levels of gender-based vulnerability residing predominantly near the Myanmar border in or near Kutupalong camp. This finding may be attributable to many variables, including the formality of settlements, longevity of those settlements, and/or relative urban/rural contexts.

Kutupalong and Nayapara in Teknaf are the two oldest official refugee camps in Bangladesh, having been established in 1992 [2]. While population size within Kutupalong has swelled exponentially since 2017, the formality and longevity of this vast settlement likely potentiate organizations to provide resources and services by providing preexistent infrastructure when compared to more informal, less established settlements. With over 600,000 refugees, Kutupalong and its surrounding expansion camps also possess significant notoriety, given its label as the largest refugee camp in the world [50]. This status has likely lead to larger degrees of funding and programming for Rohingya refugees, in general, but also for women and girls, specifically.

Settlements in and around Cox’s Bazar town are predominantly designated as ‘informal,’ with Rohingya often living in and amongst the host community. With over 250,000 people in the metropolitan area, Rohingya refugees in this urban environment likely face similar hardships as nearly half the world’s refugee population. Outside of formal, often rural, settlements, refugees confront unique challenges such as difficulty accessing services, uncertain legal status and subsequent harassment, obstacles regarding identifying and maintaining non-exploitive livelihoods, exclusion from social security systems and community support mechanisms, and discrimination [51]. Given that these threats to wellbeing affect all refugees, the finding of high levels of gender-based vulnerability surrounding Cox’s Bazar town are predictable.

Outliers of vulnerability are understandably identified within clusters of high- or low-gender-based vulnerability. Sites identified as low vulnerability outliers, such as Dhokkin, Somitapara, and Pahartoli in the Cox’s Bazaar township region, and the alternately high vulnerability outliers found in the settlements near Kutupalong and southwards, are in need of critical mixed-methods investigation. Identifying variables that influence access to resources, humanitarian programmatic impact, and security will undoubtedly shed valuable light on how these settlements have become outliers, and how best to improve gender-based vulnerability in both present and future programming.

In this study, security and health variables appear to be the predominant drivers of gender-based vulnerability in this region. Security was evidenced as a key component to both high and low gender-based vulnerability clusters in Cox’s Bazar township and near the border, respectively. It is feasible that the high level of security-based vulnerability in Cox’s Bazar township is likely secondary to settlements existing within a metropolitan area where crime levels are higher than in rural communities. But by digging into the security variables in this study, it is also apparent that women-friendly spaces are largely absent in refugee communities in this region. Women-friendly spaces not only provide safety but also act as nidus of information and support for women and girls that ‘expand protective peer networks and social assets’ [9]. Efforts to increase these spaces for security and empowerment specifically in the Cox’s Bazar township could significantly impact the well-being of women and girls.

Research has shown that WASH interventions are particularly critical to gender-based vulnerability and addressing the risk of gender-based violence (GBV) [4]. Without access to safe and private toilets, women often resort to coping mechanisms such as voluntary dehydration, urinating inside shelters, and delayed micturition, which compromise not only the health of the individual but the community at large. In this study, Hakimpara and several settlements near Nhila were identified as highly vulnerable clusters based upon WASH indicators, and could be targets of improved access to water, safe toileting options, and hygiene interventions to decrease the prevalence of diarrhea.

While these are only two examples of actionable information provided by this methodology, the potential for improving resource-allocation and cost-effectiveness of women’s refugee programming is evident and can be utilized in by diverse actors. Organizations oriented towards sector-specific activities, such as WASH, can focus on geographically-specific communities identified by disaggregated results; agencies who take a holistic approach to gender-focused aid can hone in on the composite vulnerability analysis, and researchers can utilize outlier analysis for sampling purposes. And, as more open-source data becomes available, increasingly accurate and granular conclusions can be derived. Lastly, while oriented specifically towards the Rohingya refugee community in Bangladesh, this methodology has the capacity to be applied to any humanitarian context, either utilizing these specific indicators or other context-specific variables that are likely to affect the vulnerability of women and girls. The adoption of this methodological framework on a global scale has the capacity to standardize humanitarian needs assessment procedures and subsequent programming in a statistically-rigorous, potentially cost-effective way.

### Limitations

The utilization of Pareto ranking in determining gender-based vulnerability provides only relative vulnerability rankings as opposed to absolute values. Given the aforementioned concerns regarding other means of aggregating indices variables and the aims of this study to identify the ‘most vulnerable’ communities for women and girls within the Rohingya refugee population, the researchers deemed this methodology appropriate. However, it does produce results that can be only internally validated and not absolute vulnerability scores that can be compared to alternate communities.

Furthermore, the use of inverse distance weighted spatial autocorrelation methods are likely to be dominated by nearby pairs of observations. Both number of nearest neighbors and distance threshold weighted matrices were explored during post hoc validation of the results and provided different outcomes. However, given the arbitrary nature of defining nearest neighbors and distance thresholds, and the heterogeneity of the refugee settlements, these results were omitted from this article.

Lastly, as with all vulnerability indices, this framework is subject to the critique of being too positivistic and reductionist. Previous gender-based vulnerability indices, such as the one conceived and implemented by Plan India [40] identified nine thematic indicators of gender-based vulnerability, including (1) safety and protection, (2) health and survival, (3) illiteracy, (4) poverty, (5) policy framework and implementation, (6) climate change and migration, (7) digitalization vulnerabilities, (8) cultural and social practices, and (9) urban/rural vulnerability. In the Rohingya community, cultural and social norms and preexistent systems of power are critical to understanding women’s degree of vulnerability. Limitations on their involvement in certain parts of civic and public life, ‘purdah’ or gender segregation, mobility constraints, and decision-making inequalities [52] are likely to exacerbate vulnerability in the refugee context. Given these societal norms, many Rohingya refugee women are considered to be living in ‘extremely vulnerable conditions of insecurity’ as heads of households. Furthermore, Rohingya women are often subjected to harassment, economic deprivations, and psychological, physical, and sexual violence.

While clearly important to understanding the vulnerability of Rohingya women and girls, disambiguated data were not available for many of these variables. According to the Women’s Refugee Commission [9], gender equality and women’s empowerment indicators are being monitored through the 2019 Rohingya refugee Joint Response Plan, but these data have yet to be made open-source, and there is ‘currently no structured process for analysis.’ So, similar to the Plan India gender vulnerability index in which nine indicators were ultimately pared down to four due to a paucity of data, this study’s conceptual framework was hampered by access to data. However, the index and methodology utilized in this study have the capacity to include additional parameters given the availability of future data if it is spatially codified.

### Upcycling data

The utilization of open-source data is fraught with challenges that undermine scientific rigor, accuracy, and external validation. Indicators used by organizations collecting data may be inappropriate, incomplete, or adjacent to those critical to the research question at hand. Data collection methodologies may not follow standardized or evidenced-based methods, be they sampling methodology, best practices for survey or interview conduct, or precise geo-coding. Data collection methodologies may include processes that lead to bias. Appreciating, accounting for, and being transparent about these challenges is critical to the utilization of any research that uses secondary data. Humanitarian data is certainly rife with these issues, and the imperfections of these data are recognized. However, upcycling pre-existent, humanitarian data not only minimizes the resources required for situational awareness and critical programmatic design, but also reduces the impact on the community and the potential for harm reduction [53]. In an attempt to capitalize on these resources, this study chose to utilize secondary data in an effort to demonstrate that actionable conclusions can be produced through a methodology that intentionally combines pre-existent, freely available, open-source data.

### Validation

The validation of such a methodology will prove difficult. As previously discussed, no specific construct has been academically validated to quantify gender-based vulnerability. More prudent, however, is the impact of such a method on funding, programming, and the improvement of the lives of refugee women and girls. In alternative vulnerability indices, such as the Heat Vulnerability Index [14] and the Center for Disease Control’s Social Vulnerability Index [16], success has been measured by including independent variables such as morbidity and mortality, or the ability to recover from external hazards, respectively. Unfortunately, there continues to be equipoise regarding indicators that should be utilized to gauge success regarding gender-based vulnerability within refugee communities. Future research is required to understand how vulnerability indices, and specifically gender-based vulnerability indices in refugee populations, impact not only programming but the health and wellbeing of refugee women and girls.

## Conclusion

In this study, we propose and implement a methodology for utilizing previously captured humanitarian data to identify and spatially characterize populations in need. While specifically targeted at women and girls in the Rohingya refugee community in Bangladesh, the application of a methodology that combines vulnerability indices, Pareto ranking, and spatial analysis represents a novel technique to upcycle precious humanitarian data that may be applied to many populations defined as ‘vulnerable’. While validation is critical and difficult, by leveraging such a methodology, researchers and humanitarian actors can harness the potential of open-source data to bypass the resource-intensive process of primary data collection and create actionable outcomes to target research and programming, monitor progress, and strengthen coordination and resource-allocation in many humanitarian spheres.

## Data Availability

The datasets analyzed during the current study are available in the Humanitarian Data Exchange repository, https://data.humdata.org/dataset.

## Abbreviations

GBV: Gender-based violence
GPS: Global positioning system
HDX: Humanitarian Data Exchange
IOM: International Organization for Migration
ISCG: Inter Sector Coordination Group in Bangladesh
Km: Kilometer
LISA: Local Indicators of Spatial Association
NPM: Needs and Population Monitoring
OCHA: United Nations Office for the Coordination of Humanitarian Affairs
UNFPA: United Nations Population Fund
USD: United States Dollar
WASH: Water, Sanitation, and Hygiene
